# Burden and changes in HIV/AIDS morbidity and mortality in Southern Africa Development Community Countries, 1990–2017

**DOI:** 10.1186/s12889-020-08988-9

**Published:** 2020-06-05

**Authors:** Philimon N. Gona, Clara M. Gona, Suha Ballout, Sowmya R. Rao, Ruth Kimokoti, Chabila C. Mapoma, Ali H. Mokdad

**Affiliations:** 1 College of Nursing & Health Sciences, University of Massachusetts Boston, 100 Morrissey Boulevard, Boston, MA 02125 USA; 2 Department of Nursing, MGH Institute for Health Professions, Boston, MA USA; 3 Department of Global Health, Boston University School of Public Health, Boston, MA USA; 4 Department of Nutrition, Simmons College, Boston, MA USA; 5 Department of Population Studies, University of Zambia, Lusaka, Zambia; 6 Institute for Health Metrics and Evaluation, University of Washington, Seattle, WA USA

**Keywords:** HIV/AIDS; mortality, Morbidity; DALYs, Trends, SADC countries

## Abstract

**Background:**

The 16 Southern Africa Development Community (SADC) countries remain the epicentre of the HIV/AIDS epidemic with the largest number of people living with HIV/AIDS. Anti-retroviral treatment (ART) has improved survival and prevention of mother-to-child transmission (PMTCT) of HIV, but the disease remains a serious cause of mortality. We conducted a descriptive epidemiological analysis of HIV/AIDS burden for the 16 SADC countries using secondary data from the Global Burden of Diseases, Injuries and Risk Factor (GBD) Study.

**Methods:**

The GBD study is a systematic, scientific effort by the Institute for Health Metrics and Evaluation (IHME) to quantify the comparative magnitude of health loss due to diseases, injuries, and risk factors by age, sex, and geographies for specific points in time. We analyzed the following outcomes: mortality, years of life lost (YLLs), years lived with disability (YLDs), and disability-adjusted life-years (DALYs) due to HIV/AIDS for SADC. Input data for GBD was extracted from censuses, household surveys, civil registration and vital statistics, disease registries, health service utilisation, disease notifications, and other sources. Country- and cause-specific HIV/AIDS-related death rates were calculated using the Cause of Death Ensemble model (CODEm) and spatiotemporal Gaussian process regression (ST-GPR). Deaths were multiplied by standard life expectancy at each age-group to calculate YLLs. Cause-specific mortality was estimated using a Bayesian meta-regression modelling tool, DisMod-MR. Prevalence estimates were multiplied by disability weights for mutually exclusive sequelae of diseases to calculate YLDs. Crude and age-adjusted rates per 100,000 population and changes between 1990 and 2017 were determined for each country.

**Results:**

In 2017, HIV/AIDS caused 336,175 deaths overall in SADC countries, and more than 20 million DALYs. This corresponds to a 3-fold increase from 113,631 deaths (6,915,170 DALYs) in 1990. The five leading countries with the proportion of deaths attributable to HIV/AIDS in 2017 were Botswana at the top with 28.7% (95% UI; 23.7–35.2), followed by South Africa 28.5% (25.8–31.6), Lesotho, 25.1% (21.2–30.4), eSwatini 24.8% (21.3–28.6), and Mozambique 24.2% (20.6–29.3). The five countries had relative attributable deaths that were at least 14 times greater than the global burden of 1.7% (1.6–1.8). Similar patterns were observed with YLDs, YLLs, and DALYs. Comoros, Seychelles and Mauritius were on the lower end, with attributable proportions less than 1%, below the global proportion.

**Conclusions:**

Great progress in reducing HIV/AIDS burden has been achieved since the peak but more needs to be done. The post-2005 decline is attributed to PMTCT of HIV, resources provided through the US President’s Emergency Plan For AIDS Relief (PEPFAR), and behavioural change. The five countries with the highest burden of HIV/AIDS as measured by proportion of death attributed to HIV/AIDS and age-standardized mortaility rate were Botswana, South Africa, Lesotho, eSwatini, and Mozambique. SADC countries should cooperate, work with donors, and embrace the UN Fast-Track approach, which calls for frontloading investment from domestic or other sources to prevent and treat HIV/AIDS. Robust tracking, testing, and early treatment are required, as well as refinement of individual treatment strategies for transient individuals in the region.

## Background

We sought to determine HIV/AIDs related morbidity and mortality trends from 1990 to 2017. We assessed morbidity and mortality in the 16 SADC countries using a descriptive epidemiological analysis of HIV/AIDS burden based on secondary data from GBD study in 1990, 2005, 2010, and 2017. We used secondary data from the GBD study. Examining time trends of HIV/AIDS morbidity and HIV/AIDS mortality enable comparisons across the 16 countries to understand the changing burden facing the SADC population to support policy and programmatic development in the region.

The United Nations (UN) Fast-Track or “90–90–90” approach to combatting the worldwide HIV/AIDS epidemic calls for 90% of people living with HIV knowing their status, 90% of people who know their status receiving treatment, and 90% of people on HIV/AIDS treatment having a suppressed viral load by 2020 [1, 2]. The second phase of the approach calls for upgrading the framework to 95–95-95 by 2030 [1]. HIV/AIDS-related deaths more than halved since the peak in 2004. In 2017, approximately 940,000 people died from the disease worldwide, compared to 1.9 million in 2004 and 1.4 million in 2010 [3]. In 2017, approximately 1.8 million new HIV infections occurred, compared to 3.4 million in 1996 [3]. A better understanding of the long-term trends in HIV/AIDS-related morbidity and mortality is needed to enable continued improvements on the impact of ongoing HIV/AIDS treatment programs [4, 5].

**Fig. 1:**
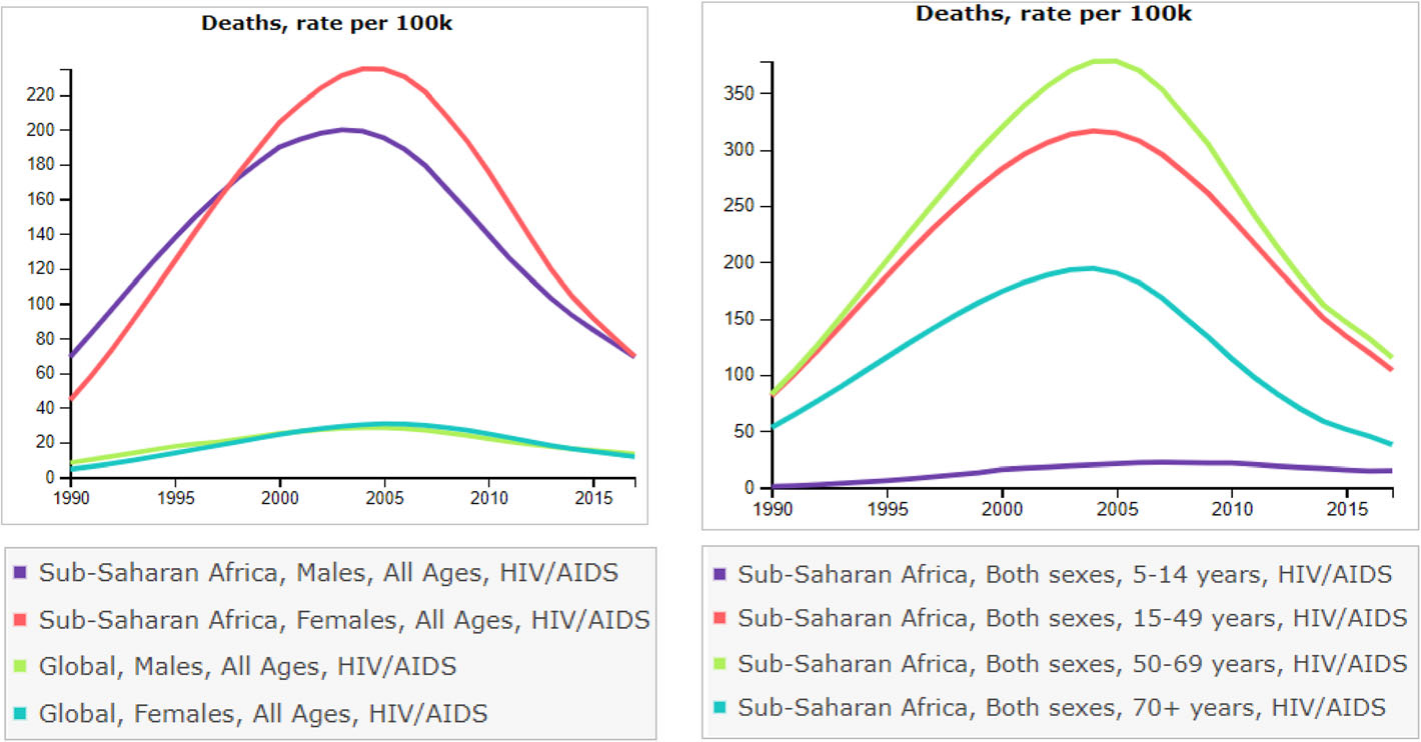
Trends of HIV/AIDS mortality in sub-Saharan Africa, 1990–2017

Sub-Saharan Africa (SSA), with more than 1 billion people, is the epicenter of the HIV/AIDS pandemic. The 16 Southern African Development Community (SADC) countries comprise ground-zero of the pandemic, with prevalence in eight countries exceeding 10% in 2015 [6]. While incidence has progressively declined since the mid-1990s, HIV/AIDS morbidity and mortality nonetheless continued to increase (see Fig. 1 right panel), reaching a peak in 2005 [3, 7]. Between 2000 and 2015 incidence of HIV declined by 35% worldwide. There was an estimated 34% fewer HIV/AIDS-related deaths in SSA in 2014 versus 2000 [8].

Despite the gains SADC countries have the highest morbidity of HIV/AIDS, with approximately 26 million people living with the disease in 2015 [9]. Of all people living with HIV/AIDS worldwide at the peak of the epidemic 2009, 34% resided in ten SADC countries, making HIV/AIDS the leading cause of death [6]. HIV/AIDS-related mortality in Southern SSA increased from being ranked 6th in 1990 to 1st in 2017; in Eastern SSA countries the ranking increased from 7th to 3rd [3, 9]. .While great progress has been achieved since 2005 to 2013, with a decline in, the number of HIV/AIDS-related deaths globally by 39%, SADC countries accounted for nearly 3 in 4 of all people dying from HIV/AIDS-related causes in 2013 [10]. HIV/AIDS, therefore, remains a massive public health threat in the region. Timely and robust evidence on mortality and trends are essential to informing policy and goal setting, program evaluation, and decision-making. Such assessment is an essential starting point for informed health policy debate to measure progress in achieving the 2030 United Nations (UN) health-related Strategic Development Goals (SDGs) [3, 11, 12] and UN Fast-Track approaches “90–90-90” and “95–95-95”.

## Methods

The GBD is a systematic, scientific effort by the Institute for Health Metrics and Evaluation (IHME) to quantify the comparative magnitude of health loss due to diseases, injuries, and risk factors by age, sex, and geographies for specific points in time. The GBD study estimates country-specific incidence, prevalence, mortality, years of life lost (YLLs), years lived with disability (YLDs), and disability-adjusted life-years (DALYs) due to diseases such as HIV/AIDS. Input data were extracted from censuses, household surveys, civil registration and vital statistics, disease registries, health service utilization, disease notifications, and other sources. Cause-specific crude and age-standardized death rates per 100,000 population were obtained from the Cause of Death Ensemble model (CODEm) and spatiotemporal Gaussian process regression (ST-GPR). Deaths were multiplied by standard life expectancy at each 5-year age-group to calculate YLLs. Cause-specific mortality was estimated using a Bayesian meta-regression modelling tool, DisMod-MR. Prevalence estimates were multiplied by disability weights for mutually exclusive sequelae of diseases to calculate YLDs [4]. YLLs were calculated using the product of age-specific life expectancy from the reference life table used in the GBD study. YLDs were calculated as a product of the prevalence of HIV/AIDS and the disability weights used to quantify health levels associated HIV/AIDS [13, 14].

Case definition for HIV/AIDS used in the GBD 2017 and comprehensive details for the methodology and modeling processes for HIV/AIDS are provided in Supplementary appendix 1, page 47 www.thelancet.com/journals/lancet/article/PIIS0140-6736(18)32203-7/fulltext#seccestitle540 [1, 4]. All GBD estimates adhere to the 14 Guidelines on Accurate and Transparent Health Estimate Reporting (GATHER). GATHER recommends making available statistical code, details on why some sources are used and others are not, and how primary data are adjusted. Methodology underlying distinct differences in estimation among UNAIDS, WHO and IHME are provided [3].

.The HIV/AIDS-related outcomes were assessed for each country in SADC, a regional economic community whose aim is to increase regional socioeconomic integration to achieve greater economic growth and poverty alleviation. Levels of development and poverty, social service delivery, and economic performance vary greatly. Nine of the 16 countries were classified in 2017 as either low or low-middle income. Only Mauritius Seychelles and South Africa were classified as high-middle income. The ability for each country to respond to the high burden of HIV/AIDS also varies considerably. SADC aims to strengthen economic cooperation and integration, providing for cross-border investment and trade, and free movement of goods and services across borders [3, 9].

## Outcome measures

The GBD results tool was used to extract sex-pooled age-standardized morbidity and mortality rates per 100,000 population for years 1990, 2005, 2010, and 2017. (available at http://ghdx.healthdata.org/gbd-results-tool).

### Deaths, YLLs and YLDs and DALYs

To facilitate comparison of HIV/AIDS outcomes of morbidity and mortality across countries, time, age-groups, and sex, the Institute for Health Metrics and Evaluation (IHME) improved previously established metrics like prevalence and incidence. How long do people live with HIV/AIDS is assessed using HIV/AIDS-specific mortality rates and HIV/AIDS-specific years YLLs. What causes people to get sick is assessed HIV/AIDS-specific YLDs which reflect the amount of time in a year that people live with a condition accounting for the severity of that condition. Adding together YLLs and YLDs yields DALYs.

To facilitate comparisons across SADC countries and eliminate potential confounding by age, outcomes are presented as age-standardized rates per 100,000 population i.e., the average of the age-specific HIV/AIDS rates weighted by country-specific proportions of a standard population in the corresponding age groups [2, 4, 5, 15].

### Change in mortality and morbidity rates over time

For changes over time, we present annualized rates of change (AROC) as the percent difference in the natural log of the in 1990 and 2017 divided by 27, i.e.,1 00*[ln(2017 Rate/1990 Rate)/27]. AROC (%)as a crude measure of linear trend over the 27-year period [4]. A positive AROC indicates an increasing trend/slope over the 27 years of HIV/AIDS, a negative AROC indicates a decreasing trend/slope.

### Expected morbidity and mortality rates in 2017

Expected rates (E) were determined using a linear equation with the country’s Socio-Demographic Index (SDI) in 2017 used as a linear predictor [16]. The SDI, which ranges from 0 to 1 is a summary measure of where a location is on the spectrum of socio-demographic development. The index is calculated from the geometric mean of three rescaled components: total fertility rate of women under 25 years of age, lag-distributed income per capita, and average educational attainment in the population > 15 years., We calculated the observed-to-expected (O/E) rate ratio.

### Uncertainty analysis

Uncertainty for each outcome was quantified using uncertainty intervals (UIs) based on 1000 bootstrap draws from the posterior distribution [16, 17]. UIs were determined by the 25th and 975th ordered values of the posterior distribution of the 1000 draws, and point estimates were computed from the mean of the draws. Changes over time were considered statistically significant when the 95% UI of the percentage change did not cross zero [3].

### Validity of mortality and morbidity outcomes

GBD uses the Joint United Nations Program on HIV and AIDS (UNAIDS) estimates as inputs in their modeling ensemble [8, 18]. For example, pediatric HIV/AIDS mortality estimates in GBD were produced with the CD4-count-specific mortality and progression parameters developed by UNAIDS [19]. Each iteration of GBD re-analyses the entire time series by use of newly available data sources from across all estimation years and continually improved methods. New data and modelling approaches effectively improve model validity and decrease uncertainty from various sources with the consequence that estimates for a given cause, location, and year might differ between GBD iterations and UNAIDS. Statistical, analytical, processing, and estimation code used to generate the GBD results are available on their website: http://ghdx.healthdata.org/gbd-2017/code (Global Burden of Disease Study 2017 (GBD 2017) - Causes of Death 5) [8].

## Results

HIV/AIDS morbidity and mortality remain major public health problems in SADC countries. Nearly all new infections in 2016 worldwide occurred in just 12 countries, four SADC countries, i.e., Mozambique, Zimbabwe, Zambia, and Tanzania. Between 2000 and 2017, HIV incidence worldwide declined by 39%, and HIV/AIDS mortality declined by 38%, but corresponding declines in SADC countries during the same period were 49 and 55%, respectively.

### Proportion (%) of deaths attributable to HIV/AIDS 2017

The five leading countries with the proportion deaths attributable to HIV/AIDS in 2017 were Botswana at the top with 28.7% (95% UI; 23.7–35.2), followed by South Africa 28.5% (25.8–31.6), Lesotho, 25.1% (21.2–30.4), eSwatini 24.8% (21.3–28.6), and Mozambique 24.2% (20.6–29.3). The five countries had relative attributable deaths that were at least 14 times greater than the global burden of 1.7% (1.6–1.8). Similar patterns were observed with YLDs, YLLs, and DALYs. On the lower end, with attributable proportions less than 1%, below the global proportion, where Comoros, Seychelles and Mauritius (Table 1).

**Table 1:** Proportion (%) of Deaths, YLDs, YLLs, and DALYs for all ages attributable to HIV/AIDS in SADC countries, 2017

Country	SDI	Deaths	AROC	YLDs	AROC	YLLs	AROC	DALYs	AROC
Angola	0.46	7.9(6.0–10.3)	13.3	1.4(0.9–2.0)	12.5	7.7(5.7–10.2)	12.9	6.3 (4.8–8.2)	12.9
Botswana	0.66	28.7(23.7–35.2)	1.2	12.4(10.9–14.1)	4.4	36.8(30.3–44.8)	0.6	28.8 (24.1–35.1)	0.9
Comoros	0.43	0.0(0.0–0.3)	12.0	0.0(0.0–0.1)	7.0	0.1 (0.0–0.4)	12.3	0.0 (0.0–0.3)	10.9
DRC	0.31	2.6(2.0–3.2)	−3.5	0.6(0.4–0.8)	− 2.2	2.7(2.1–3.4)	− 3.3	2.3(1.8–2.9)	− 3.3
Lesotho	0.49	25.1(21.2–30.4)	7.7	12.3(11.2–13.5)	8.0	32.1(27.0–39.0)	7.5	28.6(24.4–34.3)	7.5
Madagascar	0.33	1.4(1.0–2.0)	26.3	0.2(0.1–0.5)	21.8	1.4(0.9–2.2)	25.3	1.1 (0.1–1.8)	25.1
Malawi	0.35	17.0(14.6–19.8)	−0.9	6.2(5.5–7.0)	9.2	20.3(16.8–24.2)	−0.8	17.5 (14.7–20.9)	−0.7
Mauritius	0.7	0.9(0.9–1.0)	6.8	0.3(0.2–0.3)	11.4	1.8(1.7–1.9)	6.5	1.2 (1.1–1.3)	6.7
Mozambique	0.34	24.2(20.6–29.3)	8.7	7.6(6.5–9.0)	9.2	28.0(23.3–33.8)	8.8	24.5(20.7–29.5)	8.8
Namibia	0.62	23.3(18.2–29.5)	4.1	8.1(6.8–9.9)	6.0	31.1(24.1–39.0)	3.8	25.0(19.4–31.4)	4.0
Seychelles	0.74	0.8(0.7–0.9)	–	0.1(0.1–0.3)	–	1.4(1.2–1.6)	–	0.9 (0.8–1.1)	–
South Africa	0.68	28.5(25.8–31.6)	17.6	10.6(9.2–12.5)	15.8	37.7(34.3–41.5)	16.6	30.6(27.6–34.0)	16.5
eSwatini	0.58	24.8(21.3–28.6)	16.4	13.8(12.4–15.2)	15.4	30.4(26.0–34.9)	15.6	26.9 (23.4–30.4)	15.6
Tanzania	0.41	8.3(6.6–10.3)	−3.3	2.6(2.1–3.3)	−1.3	8.8(6.8–11.4)	−3.4	7.5 (5.8–9.6)	− 3.3
Zambia	0.47	18.4(15.7–21.5)	−1.5	7.0(6.2–7.8)	0.9	21.3(17.4–25.5)	−1.4	18.4 (15.4–21.8)	−1.3
Zimbabwe	0.46	13.7(11.8–16.0)	−2.7	7.8(6.8–8.8)	0.3	15.6(13.0–18.6)	−2.9	14.0 (12.0–16.5)	−2.7
Worldwide	0.70	1.7(1.6–1.8)	2.4	0.5(0.4–0.6)	4.1	3.1(2.9–3.3)	2.2	2.2 (2.0–2.4)	2.3

*YLLs* Years of life lost due to premature mortality due to HIV/AIDS, *YLDs* Years lived with disability due to HIV/AIDS, *AROC* Annualized rate of change (%) in rate per 100,000 population from 1990 to 2017, *DRC* Democratic People’s Republic of Congo, -- Data not available

#### Age-standardized mortality rate per 100,000 population in 1990, 2005, 2010 and 2017

The five leading countries with the greatest age-standardized mortality rate per 100,000 population in 2017 were Lesotho 336.6 (296.7–395.8) at the top, followed by eSwatini 245.9(222.0–280.6), South Africa 238.7(211.5–273.0), Mozambique (226.84(191.91–280.49), and Botswana 187.6 (248.7–145.6) (Table 2). These five countries had age-standardized mortality exceeding 15-fold the global mortality rate of 12.1(11.5–12.9). While the 95% UIs for eSwatini, South Africa, Mozambique, Botswana overlap, there is no substantial difference in the rates for these countries, but the rate for Lesotho is substantially higher than that for the other four since the 95% UIs do not overlap. Seychelles, Mauritius and Comoros had mortality rate lower than the global rate per 100,000. Heterogeneity in rates between countries in 2017 was high, with rate ratio between Comoros, the country with the lowest age-standardized mortality rate (0.3(0.0–2.0)), and Lesotho, the country with the highest age-standardized mortality rate (336.6((296.7–395.8) nearly 1300-fold higher. Looking back in time, in 1990, 2005, and 2010, Botswana and eSwatini had consistently the highest age-standardized mortality rates. Zimbabwe dropped out of the top 5 in 2017 (Table 2).

**Table 2:** Age-standardized Mortality Rate per 100,000 population due to HIV/AIDS in SADC countries, 1990, 2005, 2010 and 2017

Country	1990Rate (95% UI)	2005 Rate (95% UI)	2010 Rate (95% UI)	2017Rate (95% UI)	2017 Expected	O/E
Angola	1.7 (1.0–3.9)	56.7(41.6–75.4)	73.4(56.7–95.1)	68.0 (53.3–86.9)	4.7	14.6
Botswana	168.9(89.0–296.6)	979.2(777.2–1204.9)	558.1(477.4–669.5)	187.6(248.7–145.6)	1.7	111.1
Comoros	0.0 (0.00–0.1)	0.2 (0.0–1.0)	0.2(0.0–1.6)	0.3 (0.0–2.0)	5.2	0.1
DRC	66.7(46.1–93.6)	99.5(80.9–120.8)	78.7(66.5–92.7)	23.2 (18.7–28.5)	6.8	3.4
Lesotho	47.4(27.2–80.8)	1037.5(821.1–1273.6)	729.8(631.0–879.6)	336.6(296.7–395.8)	3.9	85.3
Madagascar	0.0 (0.0–0.0)	15.1(10.6–21.2)	16.9(13.8–20.6)	12.1 (9.7–17.2)	7.4	1.6
Malawi	186.3(110.1–306.0)	907.1 (753.5–1071.0)	534.3(478.2–609.7)	141.6(124.0–162.8)	7.1	20.1
Mauritius	1.3 (1.2–1.3)	1.6(1.5–1.7)	4.7(4.4–5.0)	6.4 (6.1–6.8)	1.3	5.0
Mozambique	22.2 (13.9–37.3)	491.1(390.9–605.9)	479.8(400.4–586.0)	226.8(191.9–280.5)	7.2	31.4
Namibia	67.5 (35.0–147.5)	696.3 (550.6–862.1)	462.7(386.8–559.5)	184.7(140.7–244.6)	2.1	86.2
Seychelles	0.6(0.4–1.1)	5.3 (5.1–5.5)	5.0(4.8–5.3)	5.0(4.5–5.6)	–	–
South Africa	2.1(1.6–2.8)	644.0(568.0–732.1)	599.9(544.6–668.1)	238.7(211.5–273.0)	1.6	147.7
eSwatini	2.7(1.7–6.8)	1172.4(923.0–1454.8)	1016.8(850.6–1231.1)	245.9(222.0–280.6)	2.6	96.4
Tanzania	163.7(106.6–248.6)	427.7 (349.4–516.8)	272.8(224.9–325.8)	63.3(51.3–79.1)	5.7	11.0
Zambia	242.1(144.0–398.3)	747.2 (630.7–886.0)	408.4(365.8–477.2)	137.8(121.9–158.2)	4.4	31.0
Zimbabwe	293.6(167.2–524.2)	1145.1(908.3–1420.0)	680.6(557.3–816.5)	130.4(113.0–160.8)	4.5	28.7
Worldwide	6.6(5.9–7.4)	29.2(28.0–30.5)	23.0 (22.0–24.0)	12.1(11.5–12.9)	–	–

*UI* Uncertainty Interval, *DRC* Democratic People’s Republic of Congo, *O/E* Observed/Expected

#### Age-standardized YLLs rate per 100,000 population in 1990, 2005, 2010 and 2017

The five countries with the highest age standardized YLLs rate per 100,000 population in 2017 were Lesotho 17,575.3 (14,963.6-21,596.5), Mozambique 12,892.7 (10,604.4-16,175.9), eSwatini 12,624.8 (11,203.5-14,227.9), South Africa 11,513.4 (10,118.8-13,270.6), and Botswana 8325.1 (6484.0-11,230.9). These rates were at least 12-fold higher than the 2017 global rate of 655.1 (617.7–696.4). Looking at whether 95% UIs overlap or not, the rates for Lesotho, Mozambique, and eSwatini were similar but were substantially lower than those for South Africa and Botswana since the UIs do not overlap. Comoros: 11.8 (0.1–90.1), Seychelles227.9 (206.3–255.8) and Mauritius 308.5 (290.3–326.9), with age-standardized YLLs rate per 100,000 population were 2 to 56-fold lower than the global rate. There was high heterogeneity in rates between countries in 2017. The rate ratio was nearly 1500-fold higher between Comoros, the country with the lowest rate, and Lesotho, the country with the highest rate. Zimbabwe dropped out of the top 5 in 2017 even though the rate was almost 10-fold the global rate in 2017. eSwatini was in the top five in 2005, 2010, and 2017 (Table 3).

**Table 3:** Age-standardized YLLs Rate per 100,000 population due to HIV/AIDS in SADC countries, 1990, 2005, 2010 and 2017

Country	1990 Rate (95% UI)	2005 Rate (95% UI)	2010 Rate (95% UI)	2017 Rate (95% UI)	Expected	O/E
Angola	84.9(48.8–203.2)	2848.5(2066.4-3793.6)	3660.0 (2758.8-4831.7)	3278.0(2506.4-4267.3)	243.5	13.5
Botswana	8349.2 (4541.4-14,465.7)	45,788.2(35,274.5-57,644.9)	25,370.6(21,035.1-31,576.3)	8325.1 (6484.0-11,230.9)	86.9	95.8
Comoros	0.6 (0.0–3.8)	7.9 (0.3–44.7)	9.8 (0.2–71.5)	11.8 (0.1–90.1)	270.8	0.04
DRC	3250.4 (2224.8-4564.2)	4866.9(3818.3-6011.7)	3855.1 (3154.3-4632.3)	1217.5 (977.2–1526.4)	349.9	3.5
Lesotho	2483.4(1505.3-4117.6)	50,401.8(39,208.6-62,925.8)	36,137.2(29,905.5-44,937.3)	17,575.3(14,963.6-21,596.5)	207.1	84.9
Madagascar	0.4 (0.0–2.2)	754.7(535.1–1066.8)	819.6(653.02–1016.0)	585.8(445.0–875.8)	379.7	1.5
Malawi	9673.5 (5883.5-15,647.6)	44,152.1 (35,863.1-53,260.1)	27,113.8(23,656.9-31,845.3)	7693.0 (6698.1-8944.6)	363.7	21.2
Mauritius	59.6 (56.2–63.2)	76.68(71.77–81.42)	228.7(215.2–242.7)	308.5 (290.3–326.9)	65.8	4.7
Mozambique	1185.3(754.3–1963.7)	24,430.8(19,368.9-30,469.1)	24,409.6(20,049.8-30,054.9)	12,892.7(10,604.4-16,175.9)	372.0	34.7
Namibia	3442.8 (1853.8-7324.6)	34,220.3 (26,770.7-43,294.0)	22,291.56(18,240.3-27,459.5)	9311.4(6974.6-12,367.4)	111.5	83.5
Seychelles	28.5(19.7–48.6)	231.2 (224.2–238.8)	221.0 (212.6–233.3)	227.9(206.3–255.8)	–	–
South Africa	126.8(98.5–163.9)	32,150.6 (28,206.2-36,656.7)	28,770.7(25,897.56-32,310.4)	11,513.4(10,118.8-13,270.6)	82.9	138.9
eSwatini	160.1(94.6–403.0)	57,079.8 (44,188.7-71,991.2)	49,369.6 (40,236.0-61,358.4)	12,624.8(11,203.5-14,227.9)	133.4	94.7
Tanzania	8146.7(5379.1-12,404.7)	20,370.0(16,241.1-25,110.4)	12,999.1 (10,405.0-15,890.2)	3204.4 (2575.9-3998.0)	298.5	10.7
Zambia	12,224.7(7557.4-19,670.5)	36,280.1(29,787.3-43,811.1)	20,643.5 (17,763.8-24,959.2)	7505.9 (6480.7-8866.6)	233.0	32.2
Zimbabwe	14,742.0(8549.6-25,564.8)	54,004.9(41,534.7-68,425.5)	32,599.6 (26,224.8-39,837.7)	6409.9 (5534.1-7535.1)	238.2	26.9
Worldwide	362.3 (324.9–403.2)	1579.4(1,51.4-1652.4)	1236.3 (1,18.16-1296.5)	655.1 (617.5–696.4)	–	–

*YLLs* Years of life lost due to premature mortality due to HIV/AIDS, *UI* Uncertainty Interval, *DRC* Democratic People’s Republic of Congo, *O/E* Observed/Expected, -- Data not available

#### Age-standardized YLDs rate per 100,000 population in 1990, 2005, 2010 and 2017

The five countries with the highest age standardized YLDs rate per 100,000 population in 2017 were eSwatini 1740.2(1229.0-2388.7), Lesotho 1577.3 (1117.0-2103.6), Botswana 1417.7 (991.1–1950.9), South Africa 1164.8 (819.0–1575.0), and Namibia 959.1 (639.7–1335.3). These countries hade YLDs rates at least 19-fold the 2017 global rate of 49.7(34.7–67.9). The 95% UIs reveals that while there was no substantial difference between the YLDs rate for eSwatini and Lesotho, the rates for these two countries were substantially higher than the rates for Botswana, South Africa, and Namibia. The age-standardized YLDs rate per 100,000 population for Comoros, Seychelles, Madagascar, and Mauritius were 2- to 24-fold lower than the global rate. Botswana was in the top five countries in 1990, 2005, 2010, and 2017; eSwatini and Lesotho were in top 5 in 2005, 2010, and 2017. However, Zimbabwe dropped out of the top 5 in 2017 but was part of the top 5 in 1990, 2005, and 2010. In 2017, the Comoros, with the lowest rate of 2.2, was > 790-fold smaller than eSwatini suggesting a wide heterogeneity in age-standardized YLDs rate among SADC countries (Table 4).

**Table 4:** Rate per 100,000 population YLDs due to HIV/AIDS in SADC countries, 1990, 2005, 2010 and 2017

Country	1990 Rate (95% UI)	2005 Rate (95% UI)	2010 Rate (95% UI)	2017 rate (95% UI)	Expected	O/E
Angola	5.7(2.8–14.4)	124.0 (72.6–192.4)	170.0(101.1–261.8)	180.4 (110.9–279.4)	10.0	10.1
Botswana	561.1(325.5–877.4)	2543.4(1595.0-3906.7)	1897.1 (1279.4-2736.7)	1417.7 (991.1–1950.9)	5.9	240.5
Comoros	0.6(0.3–0.8)	1.7(1.0–2.9)	1.8(1.1–3.8)	2.19(1.1–6.2)	21.1	0.1
DRC	145.6(83.4–233.6)	198.9(116.4–338.0)	160.1(93.0–270.1)	73.0 (47.4–115.1)	32.6	2.2
Lesotho	202.2(129.6–298.8)	2200.5 (1409.7-3344.8)	1978.8(1340.1-2795.9)	1577.3 (1117.0-2103.6)	14.3	110.2
Madagascar	0.1 (0.0–0.4)	34.3(20.2–53.5)	34.8(20.1–58.3)	26.2 (13.3–53.8)	40.1	0.7
Malawi	517.0 (221.3–880.0)	1955.9(1185.9-3174.2)	1329.4 (846.3–2096.1)	746.5 (526.7–1016.0)	35.6	21.0
Mauritius	1.4 (0.9–1.9)	10.4(5.3–19.3)	10.7 (8.9–30.7)	27.5 (16.9–43.0)	5.0	5.5
Mozambique	76.9(47.5–116.0)	1042.9 (662.7–1580.6)	1142.7(758.3–1678.0)	930.6 (656.9–1247.4)	37.8	24.6
Namibia	206.7(106.6–485.3)	1544.1(967.6–2316.6)	1296.3(851.5–1888.4)	959.1 (639.7–1335.3)	7.1	134.8
Seychelles	2.6 (1.5–4.5)	10.4(4.4–22.0)	10.3(4.4–21.7)	10.7 (4.3–24.7)	–	–
South Africa	17.7(12.3–23.5)	1506.1 (968.8–2229.7)	1593.5 (1039.7-2400.6)	1164.8 (819.0–1575.0)	5.7	204.3
eSwatini	29.5 (16.9–56.3)	2574.3(1674.0-3805.9)	2641.8(1775.9-3765.6)	1740.2(1229.0-2388.7)	8.4	208.4
Tanzania	456.0(267.2–709.5)	899.7(487.1–1562.1)	642.6 (371.9–1095.2)	308.5 (207.1–441.3)	24.8	12.4
Zambia	675.0(358.6–1127.0)	1655.1(1000.9-2669.2)	1181.7 (791.3–1711.3)	836.7(592.3–1118.1)	16.8	49.7
Zimbabwe	926.6(492.6–1516.0)	2458.9(1400.5-3967.37)	1688.7 (1009.5-2683.9)	957.3 (665.3–1317.3)	17.4	55.1
Worldwide	18.0 (12.6–24.7)	65.5(42.4–100.0)	60.5 (40.2–90.4)	49.7(34.7–67.9)	–	–

*YLDs* Years lived with disability due to HIV/AIDS, *DRC* Democratic People’s Republic of Congo, *O/E* Observed/Expected, -- Data not available

#### Age-standardized DALYs rate per 100,000 population DALYs 1990, 2005, 2010 and 2017

The five countries with the highest age standardized DALYs rate per 100,000 population in 2017 were Lesotho 19,152.5 (16,489.6-23,211.3), eSwatini 14,365.1 (12,817.5-16,162.5), Mozambique13,823.3 (11,501.5-17,191.3), South Africa12,678.2 (11,254.9-14,542.1), and Namibia 10,270.5 (7878.4-13,343.2). These rates were at least 14.6-fold higher than the 2017 global rate of 704.8 (662.6–747.9) per 100,000 population. Lesotho was in the top 5 in 1990, 2005, and 2017. eSwatini was in the top 5 in 2005, 2010, and 2017. Zimbabwe was in top 5 in 1990, 2005, and 2010 but not in 2017. Comoros, Seychelles, Mauritius and Madagascar had the lowest 2017 DALYs rates ranging from 1.2 to 50-fold lower than the global rate. The rate ratio between the country with the highest rate in 2017, Lesotho, vs. the country with the lowest rate, Comoros was greater than 1350-fold (Table 5).

**Table 5:** Age-standardized Rate per 100,000 population DALYs due to HIV/AIDS in SADC countries, 1990, 2005, 2010 and 2017

Country	1990 Rate (95% UI)	2005 Rate (95% UI)	2010 Rate (95% UI)	2017 rate (95% UI)	E	O/E
Angola	90.7 (53.5–207.6)	2972.4(2181.4-3902.3)	3830.0(2925.7-4983.1)	3458.4 (2665.9-4444.8)	261.4	13.2
Botswana	8910.3(5069.1-15,084.9)	48,331.6(37,560.0-59,985.4)	27,267.7(22,725.2-33,417.1)	9742.8 (7902.5-12,702.8)	92.8	105.0
Comoros	1.11 (0.39–4.4)	9.5 (1.8–46.0)	11.6(1.5–73.9)	14.0 (1.6–92.3)	291.9	0.05
DRC	3396.0(2380.6-4708.0	5065.8(4001.2-6214.9)	4015.2(3283.7-4785.2)	1290.5(1045.6-1601.7)	382.5	3.4
Lesotho	2685.6(1713.4-4330.5)	52,602.3(41,263.3-65,042.1)	38,116.1(31,909.4-46,908.7)	19,152.5 (16,489.6-23,211.3)	221.4	86.5
Madagascar	0.5 (0.1–2.3)	789.0(568.1–1109.2)	854.4(689.7–1054.5)	612.0 (475.0–913.2)	419.8	1.5
Malawi	10,190.5(6442.2-16,253.6)	46,108.0(37,922.4-54,905.7)	28,443.1(24,982.3-33,222.9)	8439.5(7398.6-9697.6)	399.3	21.1
Mauritius	61.0(57.5–64.6)	87.1 (79.5–96.9)	247.7(232.4–265.2)	336.0 (314.6–359.3)	70.7	4.8
Mozambique	1262.2 (827.5–2038.1)	25,473.7(20,321.2-31,628.4)	25,552.3(21,070.6-31,217.3)	13,823.3 (11,501.5-17,191.3)	409.8	33.7
Namibia	3649.5(2034.4-7633.0)	35,764.4(28,225.1-44,585.2)	23,587.9(28,802.8-19,514.4)	10,270.5 (7878.4-13,343.2)	118.7	86.6
Seychelles	31.1(22.2–50.7)	241.6 (231.6–254.9)	231.3(220.3–247.1)	238.5 (215.0–269.4)	–	–
South Africa	144.5(116.6–182.3)	33,656.7(29,697.4-38,266.9)	30,364.2(27,313.7-34,013.3)	12,678.2 (11,254.9-14,542.1)	88.6	143.1
eSwatini	189.6 (117.1–430.8)	59,654.1(46,749.4-74,793.5)	52,011.4(42,635.1-63,929.6)	14,365.1 (12,817.5-16,162.5)	141.7	101.4
Tanzania	8602.7(5858.7-12,863.2)	21,269.7(17,091.1-25,986.9)	13,641.7(11,024.0-16,553.5)	3512.9 (2867.9-4313.1)	323.3	10.9
Zambia	12,899.7(8203.1-20,444.4)	37,935.2(31,499.6-45,591.0)	21,825.2(18,919.4-26,217.5)	8342.5 (7258.3-9759.5)	249.8	33.4
Zimbabwe	15,668.6 (9471.4-26,524.4)	56,463.8(70,727.6-44,242.1)	34,288.3(27,593.2-41,557.0)	7367.2 (6420.4-8507.2)	255.6	28.8
Worldwide	380.2 (342.5–422.1)	1644.9(1725.6-1572.7)	1296.8(1236.0-1360.2)	704.8 (662.6–747.9)	–	–

*DALYs* Disability-Adjusted Life Years due to HIV/AIDS, *DRC* Democratic People’s Republic of Congo, *O/E* Observed/Expected, -- Data not available

### Change in mortality and morbidity rates between 1990 and 2017

**Fig. 2:**
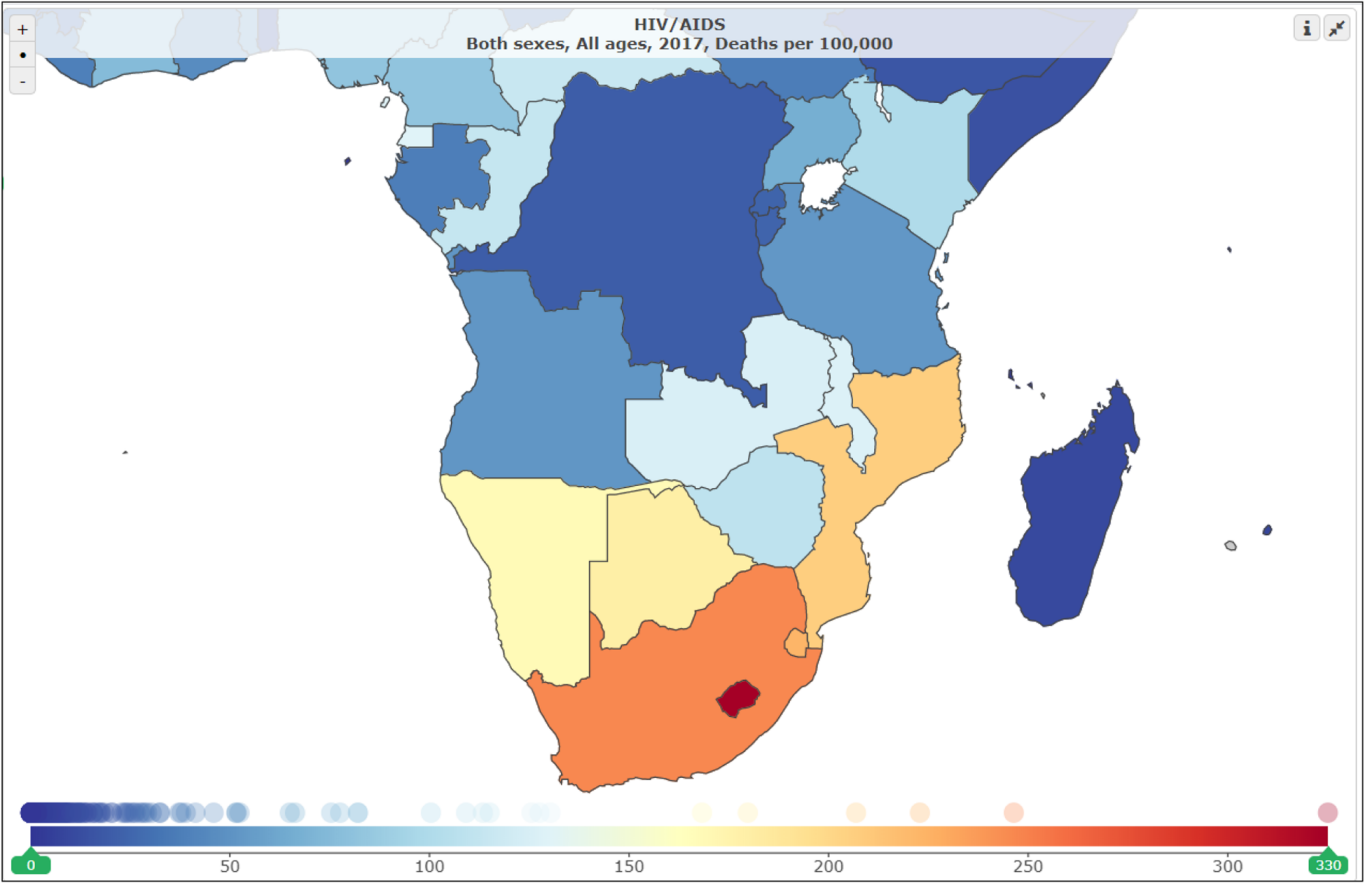
Map showing annual % change in HIV-associated death rate for males and females, 1990 to 2017

The map on Fig. 2 shows the annual percent changes in HIV-associated mortality for males and females from 1990 to 2017. Table 1 displays the 27-year annualized rate of change in mortality from 1990 to 2017 was + 2.4%, 10 countries experienced double digit slopes: Madagascar (26.3%), South Africa (17.6%), eSwatini (16.4%), Angola (13.3%), and Comoros (12.0%). The DRC, Tanzania, Zimbabwe, and Zambia experienced negative slopes. Similar patterns were observed with annualized changes in rates of YLDs, YLLs, and DALYs.

### Expected rates and ratio of observed to expected

For each metric, the SDI, a measure of where the country is on the spectrum of development based on income, education, and fertility was used to predict the expected rate of age-standardized mortality, YLLs, YLDs, and DALYs rate per 100,000 population. There was huge disparity between 2017 observed versus expected rates of mortality and morbidity in all countries except Comoros. South Africa, Botswana, eSwatini, Namibia, and Lesotho, the top five countries with highest mortality and morbidity had O/E ratios ranging from 85.3 to 147.7 for age-standardized mortality (Table 2). Similar O/E ratios in the top countries were observed with respect to rates for YLLs (Table 3), YLDs (Table 4) and DALYs (Table 5).

**Fig. 3:**
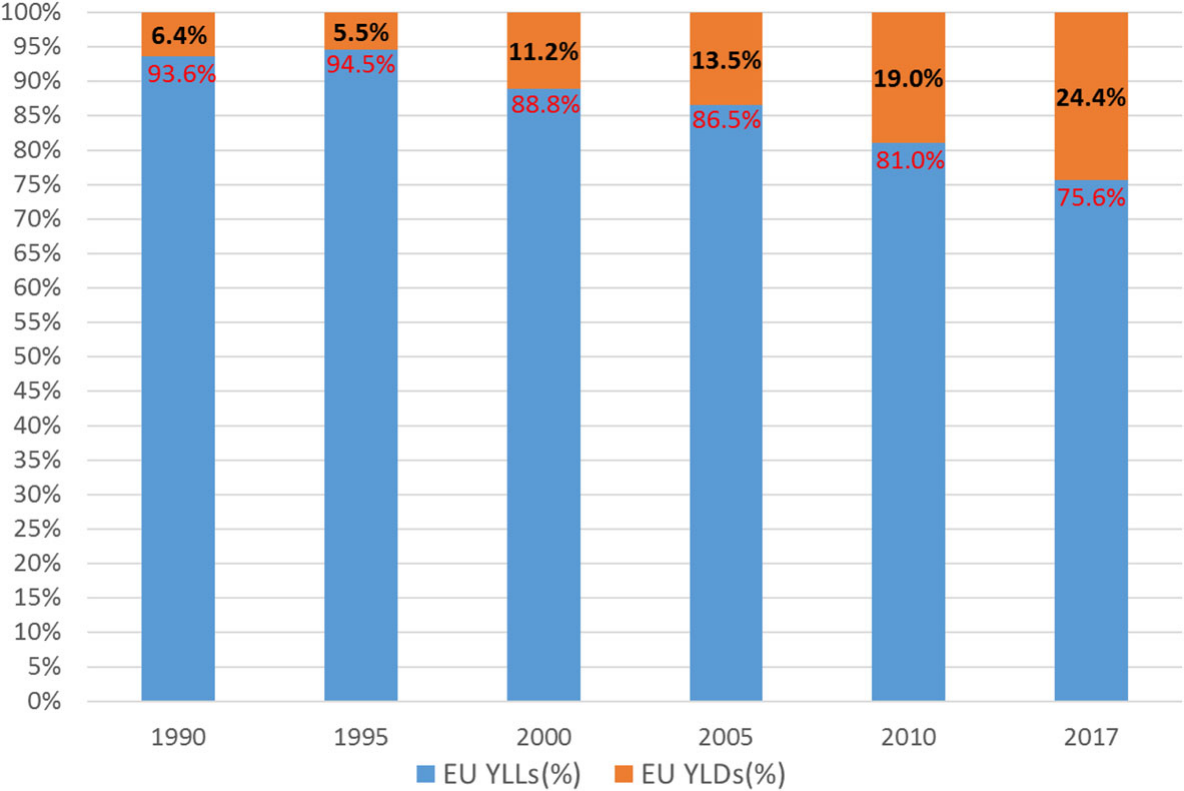
Ratio of YLLs and YLDs to DALYs attributable to HIV/AIDS in SADC Countries 1990, 2005, 2010 and 2017

**Fig. 4:**
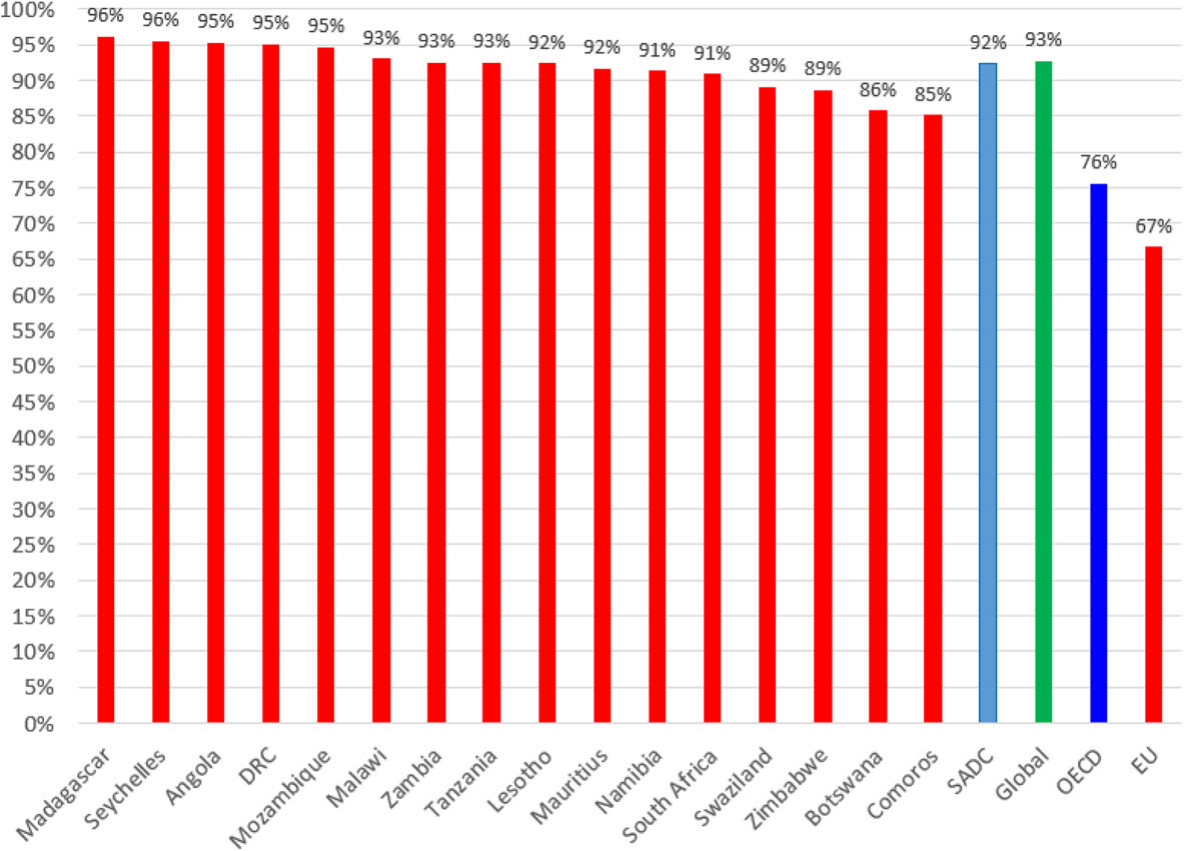
Ratio of YLLs to DALYs attributable to HIV/AIDS in SADC Countries, Worldwide, OECD, and EU 2017

To gain better perspective on the YLLs percentage for SADC countries, the corresponding YLLs percentages were 75.6 and 66.7% in the 34 member states of the Organisation for Economic Co-operation and Development (OECD) and the 28 member states of the European Union (EU) (30–40% deficit), respectively. Figure 3 displays the ratio of YLLs to YLDs as proportions of DALYs attributable to HIV/AIDS in SADC Countries, Worldwide, OECD, and EU 2017. More dramatically from Fig. 3, and highlighting the heterogeneity of the changes in the burden among rich and poor countries, the levels of YLLs proportions of DALYs due to HIV/AIDS in SADC countries in 2017 were equal to the 1990 levels in OECD and EU countries, several years before the advent and widespread use of highly active antiretroviral treatment (HAART). Botswana, South Africa, Lesotho, eSwatini, Mozambique, and Namibia all had increasing (worsening) burden, with AROC ranging from + 1.2% in Botswana to + 26.3% in Madagascar, suggesting that in these countries, the burden of HIV/AIDS has not abated, but has worsened compared to the levels in 1990.(Fig. 4).

## Discussion

We analyzed mortality and morbidity due to HIV/AIDS in 16 SADC countries between 1990 and 2017 using estimates from the GBD study. The five leading countries with the proportion deaths attributable to HIV/AIDS in 2017 were Botswana, South Africa, Lesotho, eSwatini, and Mozambique, also had the highest age-standardized mortality, YLL, YLD rates. Botswana, eSwatini, and Lesotho were among the top five countries with highest mortality and morbidity in 1990, 2005, 2010, and 2017. Comoros, Seychelles, Mauritius and Madagascar had the lowest rates in 2017. Double-digit increasing slopes in AROC (%) observed in 10 countries is worrisome. Indicating significant risk that the progress made in slowing the HIV epidemic could be reversed without a continued robust investment in health. While the negative AROC (%) in four countries, DRC, Tanzania, Zimbabwe, and Zambia is encouraging, the AROC (%) observed in Madagascar, South Africa, and Angola are concerning. While most SADC countries, except for Comoros, Seychelles, Madagascar and Mauritius had morbidity and mortality rates in 2017 greater than the global rate, there was substantial heterogeneity among the countries. The disparity in rates, measured using rate ratios, between the lowest rates observed in Comoros, and highest rates observed in Lesotho exceeded 1300-fold in 2017 suggesting that SADC countries are on very diverse trajectories regarding the burden of HIV/AIDS.

While ART has extended life for most people living with HIV, it is sobering that two-thirds of HIV/AIDS-related deaths in LMICs occurred in individuals not on ART [20]. Loss to follow-up from care and defaulting, especially for first-line treatment, significantly affect survivability. Ideally, when one defaults on the first line, the next step would be initiation into the second line, which because of cost, is out of reach for most rural communities in the SADC, thereby compromising survival of patients [21, 22].

Our most poignant finding is that the ratio of HIV/AIDS-related YLLs/DALYs of 93.6% for OECDs in 1990 (Fig. 3, first bar) is nearly equivalent but smaller, at 92.0%, than the HIV/AIDS-related ratio of YLLs/DALYs in SADC countries nearly three decades later in 2017 (Fig. 4, SADC bar) [23]. It is astounding that the 2017 ratio for SADC was lower than the ratio experienced in OECD member states 30 years prior, a period during which there was no widespread use of ART or secondary prophylaxis against opportunistic infections? Despite the advent of potent ART, SADC still lags by almost 30 years demonstrating the uneven progress that has been achieved in different regions. Notably, UNAIDS endorsed the concept of “undetectable” = “untransmittable” based on strong scientific evidence that HIV is not sexually transmitted from people living with HIV/AIDS to their HIV-negative partner if the partner HIV-positive continues to take effective ART and is virally suppressed [24–27]. Having many infected people not on treatment increases the risks for infection to the general population. Ensuring those who are infected are virally suppressed is a powerful tool to improve survival for those infected and prevent new infections.

It has been argued that HIV/AIDS has remained a massive public health threat, but global financing has plateaued, domestic health spending has stayed low among high-burden countries, and the disease incidence has not declined as quickly in younger as in older populations [20]. eSwatini, Botswana, and Lesotho had among the highest mortality rates in the world before the downward shift of the world epidemic since 2005, suggesting that the extremely high rates during the peak in 2005 continue to drive the epidemic decades later. The 2017 mortality rates in eSwatini and Lesotho remain among the highest in the world, exceeding 200 more than a decade after the global decline. Our study showed HIV/AIDS caused more YLLs than YLDs at all times, underscoring that in SADC countries survival following HIV infection is very short. It is desirable to decrease the proportion of YLLs contributing to DALYs so that patients live longer. One likely explanation of the relatively small proportion of YLDs to DALYs in SADC is people with HIV/AIDS present late for care after the onset of opportunistic infections, underscoring the need for early and periodic testing for HIV while their health is still intact. Strategies should be developed to ensure that more people who are unaware of their HIV status are tested and if necessary linked to care immediately. Health systems in the SADC region need improvement to help lengthen the lives of individuals with the disease and convert the burden of HIV/AIDS into mostly YLDs rather than YLLs. Premature mortality, measured using YLLs, is indicative of failure of healthcare management of HIV/AIDS cases in the region to convert the burden of HIV/AIDS into mostly YLDs, therefore extending the lives of the HIV/AIDS-affected individuals. Countries with better Healthcare Access and Quality Index have the potential to reduce future burden [28].

We detected huge disparities between the observed mortality compared to that expected based on the country’s level of SDI. Accordingly, SADC countries are relatively underperforming with respect to the expected reduction in disease burden compared to other countries of similar SDI. At current rates of decline in the burden of HIV/AIDS, SADC countries might not meet the SDGs target for the disease and are far from the UNAIDS goal of ending AIDS by 2030 [11]. Our study suggests that SADC countries have made some progress, but HIV/AIDS mortality and morbidity rates are still unacceptably high. While the global mortality and morbidity rates in 2017 were approximately doubled compared to 1990 levels, SADC countries such as South Africa had 2017 rate that was 114-times, Angola 40-times, and Mozambique 10-times the 1990 rate, increases pointing to a cascade of orders of magnitude.

In the SADC region, most people with HIV/AIDS are reliant on medications provided by sources outside of the region. Individuals between 15 and 49 years of age, the peak years of economic production, are the most affected by the epidemic [10]. Our findings, therefore, imply that SADC countries are economically and socially vulnerable. The number of people who do not know their HIV status is of concern. A pregnant woman with untreated HIV has up to a 45% chance of transmitting the virus to the baby. If the woman and their baby receive antiretroviral treatment, that risk drops to 1% [10, 29]. For HIV infection to become a rare occurrence, SADC countries should coordinate efforts to reduce new HIV infections, increasing access to HIV/AIDS treatment and care, particularly to religious minorities that discourage contact of their members with the healthcare system [30–32].

Government health spending is a primary source of funding in the health sector across the world, but in SSA, only about a third of all health spending is sourced from the government [20]. In Southern Africa, public funding for healthcare grew by only 4.5% each year between 1995 and 2015 [33]. Keeping the coverage of AIDS-related services at 2016 levels would lead to an increase in the burden of HIV/AIDS in almost all SADC countries. ART in SADC countries is available through “cost-free” programs funded by the Global Fund, PEPFAR, and corresponding governments [24, 25]. The heavily donor-funded ART programs have been a success story, but there is uncertainty about their long-term sustainability [34]. PEPFAR was the largest donor, providing $4.9 billion in 2016, followed by the Global Fund: with contributions from the UK ($645.6 million), France ($242.4 million), the Netherlands ($214.2 million), and Germany ($182.0 million). The US government recently proposed a 6% reduction in PEPFAR and Global Fund assistance [1, 34]. Any reduction in funding could have significant impact on HIV prevention and treatment, as most of the countries are dependent on these organizations for most of their HIV/AIDS programming budgets [12].

SADC countries should try to ramp up their domestic financing programs in order to reduce dependency on these other organizations/countries and be able to sustain the programs.

The UN Fast-Track framework advocates for frontloading resources required for full implementation of basic programs by investing $35.6 billion in LMICs in 2020. By 2030, the investment amount would drop to $32.8 billion, in the process averting nearly 28 million new HIV infections and 21 million AIDS-related deaths [1, 35, 36]. For Fast-Track goals to be successful in marking a transition toward ending the HIV/AIDS epidemic, sustained and intensified regional commitment by SADC countries together with the UN and the African Union over the next decade will be required.

## Limitations

Our study is subject to a few previously described limitations regarding the estimation of HIV/AIDS burden [3, 4]. Firstly, our study estimated mortality with HIV/AIDS as the underlying cause of death without accounting for deaths from other non-communicable causes among people with HIV/AIDS. Secondly, national-level estimates may obscure substantial heterogeneity at sub-national level. Thirdly, we had no access to traditional risk factors that influence transmission of HIV/AIDS such as presence of other sexually transmitted infections, stage of infection, male circumcision, and use of ART and pre-exposure prophylaxis (PrEP), therefore we could not explore the importance of these factors. Fourthly, SDI was used as a linear predictor to estimate expected rates in 2017, yet the SDI does not always exhibit a linear association with all causes of death including HIV/AIDS-related deaths. Fifthly, time lags in available data, absence of data from specific regions, age groups, or time periods, or unreliability in the data that are available or for geographical areas with the highest HIV/AIDS-related mortality can affect the precision of estimations. Sixth, because GBD results for HIV/AIDS are a combination of data and estimation, lags in data reporting mean that estimates for the most recent years rely more on the modelling process, as do estimates for locations with low levels of data completeness.

Despite these limitations, this study gives an insight on the disparities in morbidity and mortality within the SADC region. Efforts are underway to collect data at local levels to further reveal the granularity of estimates to reveal nuances hidden by aggregated data. This higher resolution will aid governments in focusing their efforts in regions with higher burden. For better resolution and to illuminate geographic inequality in HIV/AIDS burden, future analyses should use spatially resolved data at a 5 × 5-kilometer grid level. Community-level estimates can help identify where interventions and health policies will have the greatest impact by targeting the most vulnerable individuals.

## Recommendations

In the absence of a preventive vaccine, at current rates of decline in the burden of HIV/AIDS, SADC countries will not meet the UN’s health-related SDGs by 2030 or achieve the UNAIDS goal of ending AIDS by 2030 [11, 37]. Our study should help to inform decisions about policy and programs aiming to improve resource allocation and track accountability. SADC countries need to continue to ensure access and adherence to ART and strengthen behavioral interventions to prevent new infections. Early testing should be encouraged, perhaps rewarded, in order to link individuals testing positive to care early, when their immune systems are still strong, potentially increasing YLDs while reducing YLLs and preventing new infections. Eliminating HIV/AIDS will take sustained coordination across multiple health and social sectors in the region, along with adequate funding and supportive public policies. Governments in SADC countries should plan strategically as a block in efforts to eliminate the HIV/AIDS epidemic. The double-digit increasing slopes in AROC (%) observed in 10 countries indicate significant risk that the progress made in slowing the HIV epidemic could be reversed without a continued robust investment in health. It is unacceptable for governments to outsource the huge financial undertaking to outside forces. Governments should take responsibility for their people by making HIV/AIDS funding a priority. HIV/AIDS programming, including funding for ART manufacture, procurement, and distribution across the region, should comprise a significant proportion of the national budgets. SADC should expand its mission to include increasing domestic funding, collaborative licensing, and procurement and manufacture of ART. Rather than importing HIV/AIDS medications from abroad, local manufacturing and distribution of ART would guarantee seamless supply of the medications for all people in need. For this strategy to be effective, the SADC countries should gaurantee access to medications for people in transit and and make sure they receive care upon return.

The Coronavirus Disease 2019 (COVID-19) pandemic disrupted and put the world on edge forcing governments to implement, social distancing, and community containment, city lockdowns or traffic controls, measures which disrupted the continuum of HIV/AIDS care because of restricted hospital visits, SADC government and community partners should collaborate to sustain HIV service provision for people living with HIV/AIDS to avoid disruption of routine HIV services. Strategies such as dispensing ART in 3–6-month doses to meet the needs of people living with HIV and reduce facility visits would reduce disruption [38].

While our study looked at epidemiological data on the burden of HIV/AIDS in the SADC countries, GBD data does not address issues surrounding the economic impact of HIV, such as healthcare and occupational perspectives. Healthcare costs of HIV/AIDS and the occupational situation of people living with HIV/AIDS need to be discussed. In high-income countries, the trends indicate that an increasing proportion of the intermediate-age HIV-positive population will age prematurely, experiencing high rates of cardiovascular disease events, cancers, and neurocognitive impairment [39] and becoming frailer. Regarding occupational perspectives, the decreased life expectancy of HIV-positive persons may prompt this population to retire early from the labor market [40, 41]. Strategies should be developed to alleviate poverty, improve economic and financial opportunities for people with HIV/AIDS, and improve infrastructures to empower individuals with HIV/AIDS to continue with productive economic activity. GBD results are detailed and carefully researched using transparent methods but they are estimated and rely on many assumptions. To minimize the need for extrapolation, more primary data are needed from all countries where data accuracy and reliability can be poor.

## Conclusions

In nearly four decades the HIV/AIDS epidemic has changed dramatically as the virus has rapidly spread to all geographic regions. Globally, significant progress has been made in improving diagnosis and access to treatment. However, if HIV/AIDS-related mortality continues at current level in SADC, none of the countries will reach the SDG target of ending the epidemic by 2030. The downward trajectories observed elsewhere have been sluggish in the SADC regions. There is a need to strengthen existing strategies and create new ones to help end the disparity and help keep HIV/AIDS on a steeper downward trajectory. Education about HIV transmission and prevention and testing and immediate treatment of individuals who test positive, should be implemented and maintained and funded. Health ministries should increase efforts to ensure that accessible, affordable and stigma-free testing and treatment, including better access to viral load testing, is available to all people living with HIV/AIDS [24]. Additionally, pharmaceutical interventions like pre- and post-exposure prophylaxis which have changed prevention and treatment protocols for HIV/AIDS in other regions have not been fully implemented in SADC. SADC countries are facing challenges in meeting HIV/AIDS-related SDG targets; however, opportunity remains to take actions to accelerate progress by adopting regional multi-sectoral commitments and investments to help attain the SDGs for HIV by 2030, or achieve the UNAIDS goal of ending AIDS by 2030 [11, 37]. Regional coordination among SADC countries will further promote the attainment of SDGs.

## Data Availability

Data that support the findings of this study are available at: Table 1: http://ghdx.healthdata.org/gbd-results-tool?params=gbd-api-2017-permalink/088010c34dc209c6b1667f763c2626f2 Tables 2, 3, 4, 5: http://ghdx.healthdata.org/gbd-results-tool?params=gbd-api-2017-permalink/02aea83bf5cff055c91deb613a168a4b, or by request from the authors.

## Abbreviations

AIDS: Acquired Immunodeficiency Syndrome
ART: Anti-retroviral treatment
AROC: Annualized rate of change
CODEm: Cause of Death Ensemble model
COVID-19: Coronavirus Disease 2019
DALYs: Disability-adjusted life-years
E: Expected rates
EU: European Union
GATHER: Guidelines on Accurate and Transparent Health Estimate Reporting
GBD: Global Burden of Diseases, Injuries and Risk Factor Study
HAART: Highly Active antiretroviral Treatment
HIV: Human Immunodeficiency Virus
IHME: Institute for Health Metrics and Evaluation
LMICs: Low- and Middle-Income Countries
ln: Natural logarithm
O: Observed rates
O/E: Observed-to-expected rate ratio
OECD: Organization for Economic Co-operation and Development
PEPFAR: US President’s Emergency Plan For AIDS Relief
PrEP: Pre-exposure prophylaxis
PMTCT: Prevention of mother-to-child transmission of HIV
SADC: Southern Africa Development Community
SDI: Socio-Demographic Index
SDG: United Nations General Assembly 2030 Agenda for Sustainable Development Goals
SSA: Sub-Saharan Africa
ST-GPR: Spatiotemporal Gaussian process regression
UI: 95% uncertainty intervals
UN: United Nations
UNAIDS: The Joint United Nations Program on HIV/AIDS
WB: World Bank
WHO: World Health Organization
YLLs: Years of life lost
YLDs: Years lived with disability
